# The Development of Feeding Competence in Rehabilitant Orphaned Orangutans and How to Measure It

**DOI:** 10.3390/ani13132111

**Published:** 2023-06-26

**Authors:** Signe Preuschoft, Andrew J. Marshall, Lorna Scott, Siti Nur Badriyah, Melki Deus T. Purba, Erma Yuliani, Paloma Corbi, Ishak Yassir, M. Ari Wibawanto, Elfriede Kalcher-Sommersguter

**Affiliations:** 1 Ape Protection Unit, Four Paws, 22767 Hamburg, Germany; paloma.corbi@four-paws.org; 2 Yayasan Jejak Pulang, Samboja 75276, East Kalimantan, Indonesia; siti@jejak-pulang.or.id (S.N.B.); mdeuss123@gmail.com (M.D.T.P.); erma@jejak-pulang.or.id (E.Y.); 3 Department of Anthropology, Department of Ecology and Evolutionary Biology, Program in the Environment, School for Environment and Sustainability, University of Michigan, Ann Arbor, MI 48109, USA; ajmarsha@umich.edu; 4 Faculty of Biology and Psychology, Georg-August-Universität Göttingen, 37083 Göttingen, Germany; la.scott@yahoo.co.uk; 5 BPSILHK, Ministry of Environment and Forestry, Samboja 75276, East Kalimantan, Indonesia; ishak.yassir@gmail.com; 6 BKSDA Kalimantan Timur, Ministry of Environment and Forestry, Samarinda 75243, East Kalimantan, Indonesia; ariwibawanto7@gmail.com; 7 Ape Protection Unit, Four Paws International, 1150 Vienna, Austria; 8 Institute of Biology, University of Graz, 8010 Graz, Austria

**Keywords:** rehabilitation, re-introduction, orangutan, feeding ecology, learning

## Abstract

**Simple Summary:**

Free-living orangutan infants learn from their mother what to eat and where and when to find food. Rescued orangutan orphans, who lost their mother in early childhood, are deprived of this opportunity. In our rehabilitation program at the Yayasan Jejak Pulang forest school we try to compensate for this loss by immersing our orphans into a natural forest environment and by providing them with the possibility to learn from their conspecifics as well as from caregivers who model how to consume and process forest food. We evaluated this approach by investigating whether our orphans did adapt their food plants eaten and the parts of plants chosen to their availability in the forest for an observation period of three years. We found that our orphans did choose food plants and parts of plants comparable to mature free-living orangutans and appropriate to forest productivity. Accordingly, we assume that our approach does facilitate the acquisition of feeding competence of orphaned orangutans which in turn is crucial for their later re-introduction to be successful.

**Abstract:**

For critically endangered species, restorative conservation becomes increasingly important. Successful re-introduction of rescued wild orangutan orphans requires rehabilitation mimicking maternal rearing in the wild. Feeding competence—what to eat, where and when to find food—needs to be learned before re-introduction. We observed seven orphans (2–10 years old) for a period of 3 years during their rehabilitation at the Yayasan Jejak Pulang forest school. Of the 111 plant genera eaten by the orphans, 92 percent were known orangutan food plants. Five plant genera were eaten by all orphans in over 90 percent of the months within the observation period. The Fruit Availability Index (FAI) was used to predict which parts of a plant were consumed by the orphans. We found that the orphans ate primarily fruit when the FAI was high, but consumed more young leaves, cambium, and pith when FAI was low. Thus, the orphans exhibited food choices very similar to mature wild orangutans and appropriate to forest productivity. The orphans’ acquisition of feeding competence was facilitated by their immersion into a natural forest environment in combination with possibilities for observational learning from conspecifics as well as caregivers modelling food processing and consumption.

## 1. Introduction

Since the 1980s large scale land conversion has led to devastating habitat loss in Indonesia and Malaysia and has contributed to pushing orangutans to the brink of extinction [1,2,3]. Restorative conservation measures are now needed to turn the tide, and the re-introduction of rescued wild orangutans into suitable habitat is one of them. In this paper we explore how immature orangutans can be rehabilitated in such a way as to compensate for the loss of their biological mothers prior to their confiscation and rescue by authorities. 

Re-introductions of rehabilitant immature orangutans have been attempted since the 1970s, but persistent challenges must be overcome before re-introduction can be considered a viable conservation tool, e.g., [4,5,6,7,8]. Among the many things that can go wrong when orangutan orphans are raised in human care is the failure to acquire feeding skills that are appropriate to sustain the orangutan once mature and released from human care into a natural habitat. So far, rehabilitation programmes have not routinely assessed the development of feeding competence during the process of rehabilitation and prior to re-introduction. In recent years, programmes have attempted to provide post-release monitoring after re-introduction, e.g., [9,10,11,12], but by this time a large part of an individual’s learning history is already fixed and the effects of insufficient or faulty learning are almost irreversible. It is therefore vital to, on the one hand, document the development of feeding competence during the rehabilitation process and, on the other hand, act in a timely manner to correct developments that are likely to lead to problems once the orangutan is mature and released. That re-introduced orangutans can fail to adapt and become prone to get entangled in human-orangutan conflicts is well-known, although rarely documented (notable exception: [13]). The acquisition of appropriate feeding skills is only one facet that will determine whether a rehabilitation is successful. It is essential to improve our knowledge about therapeutic interventions as well as of techniques to document them, since, increasingly, the conservation of our closest relatives, the apes, will depend on the success of restorative conservation efforts.

Orangutan feeding ecology is complex because it is, particularly for Bornean orangutans, strongly influenced by supra-seasonal, irregular cycles of food scarcity and food abundance, and also because orangutans are highly adaptable, eating different plants at different locations and adapting with dietary adjustments to habitat changes, e.g., as a result of forest fires or logging, e.g., [14]. Remarkable intra-species variation therefore emerges between sites, and even between individuals living at the same site, and probably also within individuals once their habitat has been profoundly altered. A few patterns have emerged solidly from past research: Orangutans prefer large fleshy fruits, but these are rarely available, especially on Borneo [15]. For the most part, orangutans make do with less preferred fruits, and when conditions get really dire on Borneo, they live almost exclusively on leaves and cambium [16]. The term “fallback foods” (first mentioned by [17]) is applied variously to the foods eaten increasingly during periods of food scarcity and to less preferred fruits, such as figs and rattan fruit, which are small, not fleshy but occur in large clusters [18,19]. Marshall & Wrangham [20] hypothesized that one can distinguish between staple fallbacks, which are eaten almost all the time and can serve as sole food supply during periods of food scarcity, and filler fallbacks, which are only eaten during periods of food scarcity but never constitute the entire diet. It can therefore be assumed that filler fallbacks are eaten to prevent starvation, and can thus be lifesaving, while staples provide nutrients that are always needed.

The distinction between fallback foods and preferred foods and within the category of fallback foods between staple and filler foods is based on food quality [19]. It can be assumed that the ratio of nutrient to fibre and of caloric value (energy return) to feeding effort (energetic investment) are reasonable proxies of food quality [21,22]. In this way, food quality will vary between different organs of the same plant, and, likewise, between plants. Ideally, an orangutan would consume those foods that have the highest quality amongst those available at the orangutan’s location at that point in time. However, this calorie-focused notion falls short of appreciating an organism’s need to balance various nutrients, such as proteins, lipids, minerals and vitamins [23,24,25]. Food choices are made not only between food taxa, but also in regard to parts of plants, such as cambium, leaves, flower or fruits, etc. An essential component of food choices is the availability of certain types of foods, which can only be determined through systematic collection of plant phenology data.

Orangutans learn from their mothers what and how to eat. By the age of weaning an orangutan infant’s diet overlaps about 90% with that of its mother [26]. After weaning, juveniles have more social encounters with other conspecifics, and they learn about additional nutritional options from them [27]. Beyond that time window of immaturity, mature orangutans are very conservative (neophobic) in their food choices [28], although they are capable of adapting to radical ecological changes, e.g., those caused by forest fire and the resultant loss of previous dietary options. Orangutans might therefore be less conservative and potentially more neophilic/experimental in their food choices after major upheavals of their living conditions [29,30]. Rehabilitant and translocated orangutans had larger dietary breadths shortly after their re-introduction into a natural habitat, whereas the number of food items diminished once they became more familiar with the new site [12]. This observation can be explained as a result of orangutans learning to make more efficient food and ranging choices, which permits them to become more conservative as they gain experience in a new area. Furthermore, orangutans exposed to human care appear to be more neophilic than wild ones [28], which suggests that human-reared orphans might make fatal mistakes trying foods that are not healthy.

The task of teaching orphaned orangutans is daunting because orangutan habitats are characterized by extremely high plant species-richness, containing many hundreds of potential food plants and an even larger number of taxa that must be avoided. For example, a list compiling all plant species consumed by orangutans contains 1486 species in 449 genera, of which 839 species in 440 genera have been formally identified ([31], see link on p. 138 and Table 9.3). Few people possess the ability to identify all of the food and non-food plants in an orangutan’s environment, posing a major challenge to caregivers in rehabilitation programmes.

A second challenge is how to assess the extent to which orphan orangutans are learning age-appropriate feeding skills. Feeding competence is usually measured by recording all actual food choices of a focal individual made during random observation times. However, since ‘competence’ implies an evaluation, it is necessary to compare these food choices to a standard. Simply measuring food repertoire size seems not a suitable operationalisation of feeding competence, aka “the more the better”, in light of the above-mentioned reduction of variability associated with learning [12]. For wild immatures, evidently the food choices of their mothers can serve as a standard [26]. Just how much immatures depend on their mother’s modelling is highlighted by the fact that the difference in food resource usage between resident and immigrant adult females is reflected in the food choices of their offspring [26]. By contrast, the diet of orphaned immatures can only be grossly compared to an inferred diet of competent adults, such as what wild adults in a similar location are eating. High intra-species variability in diet composition complicates selection of an appropriate wild reference population. The comparison is further complicated by supra-seasonal cycles of food abundance and scarcity. Obviously, it would be best to compare orphans’ choices during food scarcity with wild orangutan’s choices during scarcity, or food abundance/masting years, respectively. However, not many studies of wild orangutans have been conducted long enough to match for both, geographic region/habitat type and El Nino cycles.

**Figure 1:**
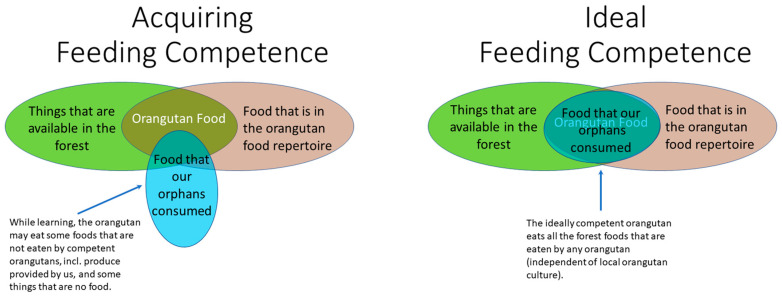
Available orangutan food items are those that both occur in the forest and are listed on “the orangutan food list” because field researchers have observed orangutans eating them. (**Left**) An orangutan still in the process of acquiring competence will not be able to utilise all the available foods and may eat some food items not on “the orangutan food list”. (**Right**) An ideally competent orangutan would eat 100% of the available food items.

Despite these challenges, an assessment of the developing feeding competence must be made during the rehabilitation process (see Figure 1) or else it might be too late to correct developmental aberrations that are bound to cause problems once the orangutan is released into the wild. Survival depends on the ability to use foods that provide sustenance when preferred foods are scarce (i.e., to find and process fallback foods). For this reason, we attempted to identify which plant genera and plant organs were utilised as fallback foods by orphaned orangutans immersed in a forest school of old secondary lowland forest. This knowledge would enable predictions about their ability to utilise foods at a later release site, by comparing the plant inventories of both sites. Identifying fallback foods will help assessing food repertoires and a rehabilitant’s likely ability to survive periods during which preferred foods are scarce.

On a pragmatic level, moreover, the identification of basic foods eaten year-round reduces the number of plant genera caregivers must learn. Instead of having to become tropical biodiversity experts, caregivers can be trained to reliably recognise a more feasible number of 20–25 plants (or plant genera). The focus on identifying basic and fallback food plants could guarantee better quality of feeding data but also enable the facilitation and modelling of eating key fallback foods for the orphans by caregivers, thus laying the base for a rehabilitation programme-specific feeding skill culture shared by caregivers who act as mothers and orphans representing the novices [32].

We investigated the development of feeding competence in seven orangutan orphans (aged between 20 months and 10 years) over 3 years while they underwent rehabilitation at a forest school of old secondary lowland forest in East Kalimantan. We aimed to document the food choices of orphaned orangutans in relation to the foods available. Specifically, we asked:

1. Did the orphans eat the same plant genera that wild orangutans eat? Did they ignore potential food sources or mistakenly consume potentially dangerous plants?
2. Were there food plants which the orangutans consumed throughout the year?
3. Which parts of plants did the orangutan orphans eat, and how did this correspond to their availability in the forest? Did orphans indeed prefer fruit and what did they eat if fruit was scarce?
4. Did orphans choose the same fallback foods that wild competent orangutans would have chosen?
5. Were there differences in dietary (taxonomic) breadth between individuals, between phenological periods, or within individuals? And what might have caused them?

## 2. Methods

### Subjects & Forest School Management

We conducted this study at the Yayasan Jejak Pulang (YJP) forest school in East Kalimantan, Indonesia. The school is currently home to 12 orphans that were confiscated by Indonesian authorities and entrusted to YJP for rehabilitation with the aim to re-introduce them to their natural habitat once they have “come of age”. Seven orphaned orangutans were present at the onset of the study and contributed data to this study (Table 1). The rehabilitants were managed in two groups with differing daily routines. The three oldest orphans are labeled “juveniles”: Amalia, Eska and Cantik spent 24 h each day in the forest from August 2019, with a 1:1 ratio of caregivers enabling them to roam alone without being lost from sight. In the first 7 months of 2019, Eska and Cantik still returned to a night cage for sleeping, but as soon as Amalia joined them, she and Cantik switched to making tree nests every night, whereas Eska wavered between sleeping in the forest and in the cage. He eventually took to sharing Amalia’s nest most nights. 

**Figure 2:**
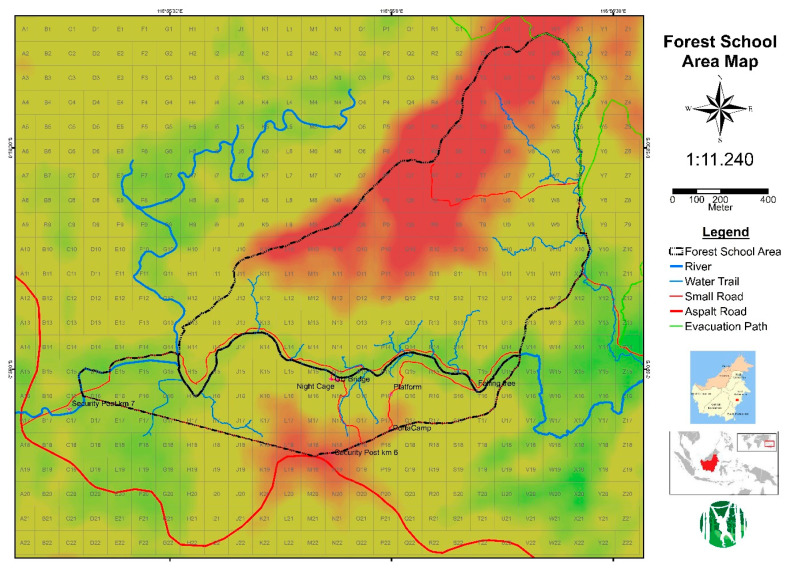
Topographic map of the Forest School. Each quadrant is 100 × 100 m in size. The forest school is outlined with black, North of the river flowing in West-East direction. Supporting facilities are South of the river, inside the patrolled perimeter.

The second group is called “weanlings”, because they reached weaning age during the study period, but were infants (Forest School level 1, FS1) when the study began. The management of these four orphans changed along with their development, but a ratio of ca. 1.3 orangutans to 1 caregiver, varying between 3 and 4 caregivers per day, applied throughout. At the beginning, they slept at the nursery and foraged during the day in the South-Eastern area of the forest school (Figure 2). From November 2020, the “weanlings” were gradually promoted to level 2, spending all daylight in the forest and sleeping in a night cage. At the same time, some caregivers of the “juveniles” were placed with these “weanlings” to direct and encourage their use of forest foods and wider roaming. From 2021, we arranged for weekly meetings between the youngest juvenile Cantik and the “weanlings” to facilitate bonding and knowledge transfer between them. Due to COVID-19 transmission prevention measures, Amalia and Eska spent some weeks encaged in August 2021. From November 2021 the “weanlings” and the “juveniles” started to occasionally socialise. By the end of 2021 all of the “weanlings” had taken to sleeping in night nests in trees by using old nests previously made by juveniles.

Orphans received supplemental food in addition to the forest foods. At ca. 5:30 the orphans received a small breakfast, e.g., 1 tomato or milk for the infants, to keep them hungry and food oriented. Between 16:00 and 17:00 dinner was offered at one of the feeding platforms in the forest school. The dinner consisted of isotonic water (milk for the infants), vegetables and a protein source, such as a boiled egg, tofu or tempeh. Fruit, such as watermelon or salak, was provided only rarely. During the fruiting season the juveniles often did not come to the platform but made nests in the fruit trees in which they had last fed.

## 3. Data Collection

### 3.1. Recording Foods Consumed by Orphans 

The first data set is based on paper-and-pencil records of every plant genus (and species, if known) eaten by an orangutan at the forest school per month. To quantify consumption, we counted how many different plant genera were eaten by the orphans. These data, compiled per orangutan and month, extend over a period of 26 months, from January 2019 to February 2021.

The second data set was collected using the software ZooMonitor [33]. Behavioural data included forest food eaten, recorded on the genus level (and species if known) and part of plant eaten. Plant parts recorded were leaf (young vs. mature), pith, inner bark (i.e., cambium), stem, fruit, seed, and flower. Forest foods eaten included both forest foods chosen by the orangutans and those consumed in the context of modelling by caregivers. For most of the months, almost all consumed forest foods were chosen by the orangutans. The proportion of forest food modelled by caregivers for the three juveniles Amalia, Eska and Cantik was on average 1.5 percent (range: 0–23% per month), and on average 2.5% (range: 0–30% per month) for the four weanlings Kartini, Tegar, Gonda and Gerhana. These data were collected in one to several one-hour sessions per day, which in total amounted to two or three full days per month and orangutan. These data are expressed as percentage of forest food eaten and span a period of 36 months (from January 2019 to December 2021).

All data were collected by caregivers. We analysed plant genera only, because not all caregivers could reliably identify plants to the level of species (for species see Table S4). We recorded but did not analyse fruit and seeds separately (as recommended by [31], because for some plants (e.g., *Macaranga gigantea*) it was impossible to discern which part of the fruit an orangutan ate, and in other cases the observer would have to have been familiar with the exact fruit anatomy in order to identify which part of a fruit was eaten.

### 3.2. Phenology Data

Phenology data were collected on 4 transects which were designed to represent the entire forest school, covering different elevations and soils [34]. Transect A comprises of 475 trees, transect B of 427 trees, transect C of 419 trees, and transect Sungai along the river of 93 trees, adding up to 1414 trees alive in February 2019. 95 trees died off during the study period, resulting in 1319 trees alive in February 2021. Phenology data include the number of trees bearing ripe fruit, unripe fruit, young leaves, and flowers. The caregivers Nor Faniyansah and Jaka Imandhana were trained by members of the BALITEK botany team, Pak Zainal Arifin and Pak Bina Swasta Sitpu, to identify plant species and collect phenology data. 

We collected phenology data in the last week of each month. Unfortunately, due to shortage of caregivers we were unable to collect data for January, March, April and October 2019, for November and December 2020, and during COVID restrictions for July and August 2021. The reproductive patterns of trees along the transects were captured in the Fruit Abundance Index [35] to distinguish relatively fruit-rich and fruit-poor periods as potential predictor of food choices. We calculated the Fruit Abundance Index (FAI) for the forest school area based on the percentage of trees fruiting within the four transects. We distinguished the availability of unripe and ripe fruit (unripe FAI—only trees with unripe fruit, and ripe FAI—only trees with ripe fruits). The Total FAI is not the sum of FAI ripe and unripe as some trees might bear ripe and unripe fruit at the same time. 

## 4. Analyses

### 4.1. Assessing Orphans’ Food Plant Repertoires: TOFL Test

We compared the plants consumed by the orphans with the food plant repertoires reported for any free-ranging orangutans. Orangutan field workers have contributed data to a comprehensive list of plants eaten by orangutans at various sites (see [31]) but note that the list has not been updated since 2007. Therefore, we also included the additional orangutan foods reported for Danum Valley, Sabah [36]. 

This data compilation yielded 449 genera of plants. Hereafter we refer to this as The Orangutan Food List (TOFL). 

**Competent choices** were defined as the intersection (*∩*) between the set of plants on the TOFL and the set of plants consumed by JP orphans in forest school (FS).

- {plants on TOFL} ∩ {plants eaten in FS} = competent food choice ALL PLANTS

**Figure 3:**
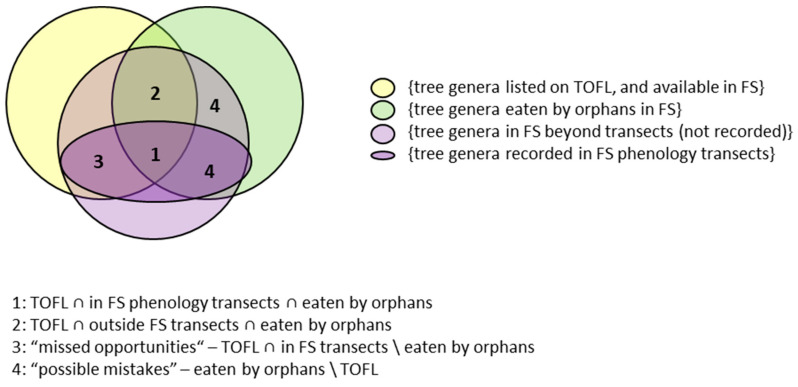
Relationships of sets used to assess competence of orphans regarding food plant choice. Relationships between the sets are indicated by mathematical symbols from set theory. ∪ signifies an intersection of sets: the elements they share in common; ∩ signifies a disjunction of sets: all elements in either set; \ signifies the difference between the first and second set, identifying the elements that belong to first but not the second set.

The plants actually consumed by the orphans were necessarily a subset of the plants available at the forest school (Figure 3: sets 1 and 2). A complication arose because not all the plants actually ingested by an orphan also occurred on the phenology transects (Figure 3: set difference indicated by 1 vs. 2).

Moreover, from the plants anywhere in the FS, including trees anywhere in FS and (∪) non-tree plants anywhere in FS, only tree genera along the transects were recorded, no other plant forms (b). From the tree genera anywhere in the FS, including trees recorded on FS transects and (∪) trees beyond transects, tree genera beyond transects are not included in the phenology data (c). Nonetheless, we need to refer to this smaller set of food opportunities to determine what proportion of the known orangutan foods the orphans consumed (d).

b. {plants anywhere in FS} = {trees anywhere in FS} U {non-tree plants anywhere in FS}
c. {trees anywhere in FS} = {trees recorded on FS phenology transects} U {trees beyond phenology transects}
d. {trees on FS phenology transects} ∩ {trees on TOFL} ∩ {trees eaten in FS} = competent food choice TREES

“**Missed opportunities**” refer to trees present on the transects but not consumed (\) by the orphans (Figure 3: set 3).

e. {trees on FS phenology transects} ∩ {trees on TOFL} \ {trees eaten in FS} = missed opportunities

“**Possible mistakes**” represent plants ingested by orphans that were not (\) on the TOFL (Figure 3: set 4)

f. {plants eaten in FS} \ {plants on TOFL} = possible mistakes

### 4.2. Plant Genera Eaten throughout the Study Period

It was recorded whether a particular plant genus was eaten in a certain month or not for each orangutan. In other words, only one entry per genus was possible, irrespective how often the genus was consumed during that month. This led to a table with plant genera in rows and individuals in the column. Since the data collection spanned 26 months, the maximum value was 26 if a genus was eaten in each month by an individual. An exception is Amalia for whom data collection covered only 20 months (Table 1). The values were then converted into % of 26 months (or 20, respectively) to enable comparisons, e. g., a genus consumed in 26 months (or 20, respectively) is expressed as 100%. This information ignores which part of a plant was eaten. Plant genera eaten in at least 80% of the months are defined as plant genera eaten throughout the year/study period (potential staple foods). Comparisons of the relative frequencies of these most often eaten plant genera were conducted between individuals by using Spearman’s rank correlations with Bonferroni corrections for multiple comparisons.

### 4.3. Phenology as a Predictor of Food Choices

We assessed if the consumption of certain parts of plants could be predicted by phenology patterns (ripe FAI, unripe FAI) using regression analyses. Furthermore, Pearson’s correlations were used to reveal changes in the relative proportions of different plant parts, esp. between fruits and young leaves, pith, and cambium in an orangutan’s diet. Proportions of individual plant parts eaten were calculated as the percentage of all parts of forest food eaten by an individual. Potential filler foods are defined as parts of plants that are consumed only during periods of food shortage. Therefore, we used the Fruit Availability Index to investigate whether the consumption of certain parts of plants increased during periods of fruit scarcity.

Individuals’ food plant repertoires (taxonomic breadth) were defined as the number of different plant genera they consumed. This was measured per month and related to phenology so that we could assess not only an individual orphan’s total food plant repertoire, but also the development of their food plant repertoire over time and in relation to forest productivity. Comparisons of individuals’ taxonomic breadth, i.e., the number of different genera eaten per month, were conducted by using Spearman’s rank correlations with Bonferroni corrections for multiple comparisons. Regression analyses were conducted to test whether phenology predicted dietary breadth. All analyses were conducted in IBM SPSS Statistics 22 and R 4.2.2 [37]. 

## 5. Results

### 5.1. Did the Orphans Eat What Wild Orangutans Eat?

The studied orphans were seen to eat 111 identified genera of plants comprising trees, herbs, lianas and shrubs (Table S1). Trees represented 83 (i.e., 75%) of these identified plant genera; herbs, lianas and shrubs summed up to 28 genera (i.e., 25%).

As a rough approximation to the feeding competence developed by orangutan orphans attending the YJP forest school, we assessed whether they consumed the same plant genera that have been recorded as orangutan foods in TOFL (see Methods; see Figure 3: intersections 1 and 2). 

102 of the 111 genera (i.e., 92%) of all identified plants were good choices, i.e., they were listed on TOFL or consumed at Danum Valley [36]. 

Among these 111 eaten plant genera were the 83 tree genera eaten by the orangutans at forest school, 64 genera were recorded along the phenology transects and 19 outside the transects. Of the 64 genera along the transects, 61 were “good choices”, because they are listed on TOFL, or were consumed by orangutans at Danum Valley [36], 3 genera were eaten but are not on TOFL (“possible mistakes”) and 42 edible genera (cf. TOFL) were not consumed (“missed opportunities”). Of the 19 genera outside the transects, 15 were “good choices”, included on TOFL (or [36]) and 4 genera were “possible mistakes” and should not have been eaten (Figure 3).

Missed opportunities refer to food trees present in the Forest School (FS) but not consumed by the orphans (Figure 3: set 3). The 42 missed opportunities represent plant genera from which primarily fruits and seeds were consumed by wild orangutans. Only 20 of these have actually produced fruit in the course of the observation period (Table S2). Therefore, the actually missed opportunities might be more appropriately represented by these 20 tree genera, i.e., that did provide fruit/seeds during the study period. 

Possible mistakes represent plants ingested by orphans that were not on the TOFL (Figure 3: set 4). Of the 111 plant genera consumed by YJP orphans 11 genera were not on the TOFL. However, of these 11 genera, *Callerya* spp. and *Callicarpa* spp. (both trees) were listed by [36] for Danum Valley, Sabah, and can therefore still be considered appropriate orangutan foods. This left 9 genera (7 tree genera—*Cananga* spp., *Melicope* spp., *Paracroton* spp., *Guioa* spp., *Caryodaphnopsis* spp., *Camellia* spp., *Phaenthus* spp.—and 2 herb genera—*Diplazium* spp., *Saccharum* spp.; see also Table S1) not eaten by free-ranging orangutans anywhere but still consumed by the orphans. These 9 genera could represent inappropriate food choices by orphans lacking both competence and competent role models (Figure 3: set 4).

In sum, orphans basically consumed the same plants that free-ranging orangutans are known to consume, but they also potentially made mistakes in 8% of their food choices, by eating potentially non-edible plants. They missed out on 20 genera of edible and available fruits.

### 5.2. Were There Food Plants That the Orangutans Ate throughout the Study?

We also tried to understand if some plant genera might serve as staple foods, irrespective of the part of the plant that was consumed.

Table 2 lists the plant genera eaten by all seven orangutans in more than 50% of months and the frequency ranking of each genus for juveniles (i.e., Amalia, Eska and Cantik) and weanlings (i.e., Kartini, Tegar, Gonda and Gerhana), respectively. It is noteworthy that the juveniles and weanlings differed in their rankings of most regularly consumed plants. The genera consumed in over 80% of the months have similar rankings in both groups. But from 80% downwards the genera are increasingly divergent between the age groups.

Of the 5 plant genera eaten most, *Ficus* spp. grows as tree, hemi-epiphyte, or liana. *Artabotrys* spp. is a liana, the herb *Calamus* spp. (rattan) also grows as a climber, the second tree, *Borassondendron* spp., is a palm, and *Alpinia* spp. is an herb. Three frequently eaten tree genera were *Artocarpus* spp., *Pternandra* spp. and *Callicarpa* spp. 

Note that bamboos were an important food source for weanlings, being eaten in 80% of the months and ranking sixth but were consumed far less by juveniles. There were some genera consumed more often by one group than by the other: *Leea* spp., *Sterculia* spp., *Stenochlaena* spp. and *Donax* spp. ranked high in weanlings but were eaten rarely or not at all by juveniles. By contrast, *Symplocos* spp., *Knema* spp., *Polyalthia* spp. and *Dillenia* spp. ranked higher in juveniles but were consumed rarely by weanlings.

**Figure 4:**
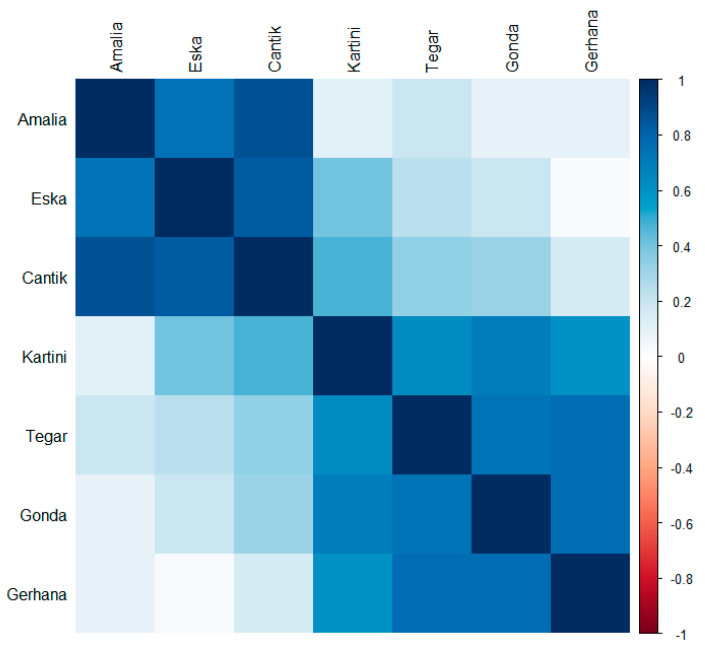
Spearman’s rank correlation matrix comparing the taxonomic breadth between individuals. N per orangutan = 26 months (except for Amalia: N = 20 months).

**Figure 5:**
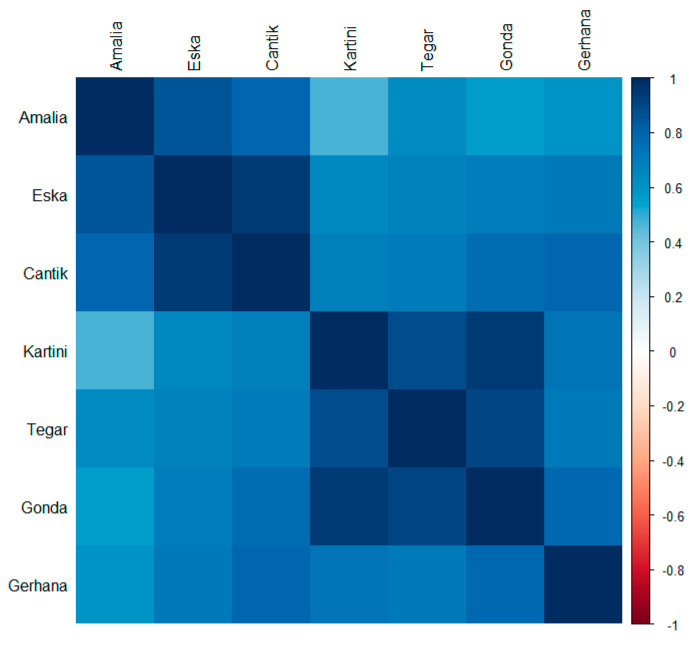
Spearman’s rank correlation matrix comparing the relative frequency of plant genera eaten between individuals. Plant genera included (N = 15) were eaten in at least 80% of the months by one of the seven individuals.

The taxonomic breadth, i.e., the number of different genera eaten per month, is more similar within the groups of juveniles and infants than it is between the different age categories (Figure 4). When comparing the relative frequencies of the most often eaten plants, i.e., those eaten in at least 80% of the months, individuals within age groups were more similar to their peers in the same age group than to those in the other age group (Figure 5). For the respective genera and percent values see Table S3.

To summarise, orphans used 9 plant genera virtually all the time. Caregivers can be trained to identify these important plants, which include lianas and herbs along with trees.

### 5.3. Forest Productivity and Choice of Plant Organs

While 92% of the plant genera (i.e., 102 out of the 111 genera) that were consumed by YJP orphans were also consumed by free-ranging orangutans, this comparison affords only a coarse approximation to feeding competence, because it ignores which parts of the plants were consumed. Information about the parts of plants consumed is important, because plant parts differ in nutrients. It is well-known that competent orangutans prefer fruit, yet it is knowledge of fallback foods that their lives depend on during periods of food scarcity. Fallback foods are usually identified as parts of plants. Feeding competence, therefore, must include having the capacity to make the right choice of plant parts dependent on the circumstances.

**Figure 6:**
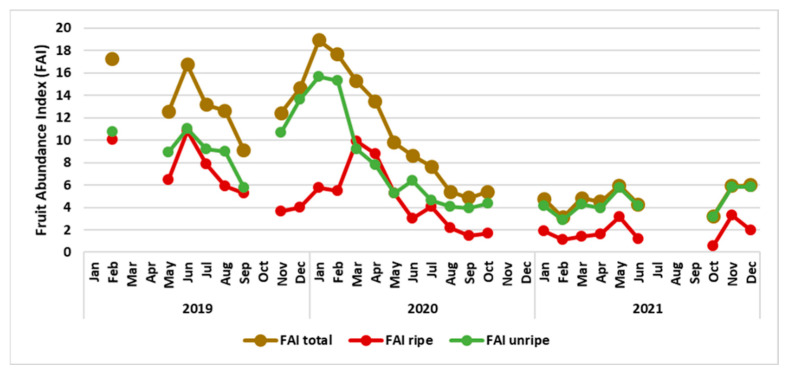
Variability in the Fruit Abundance Index (FAI) in the forest school area between January 2019 and December 2021. Gaps in the data collection were sometimes unavoidable.

As a predictor of food choices by orphaned orangutans, we calculated the Fruit Abundance Indices (Figure 6). With this information we proceeded to investigate if the orphans would eat more fruit if fruit was easily available, i.e., whether the FAI would predict the proportion of fruit in the orangutans’ consumption of plant organs. 

**Figure 7:**
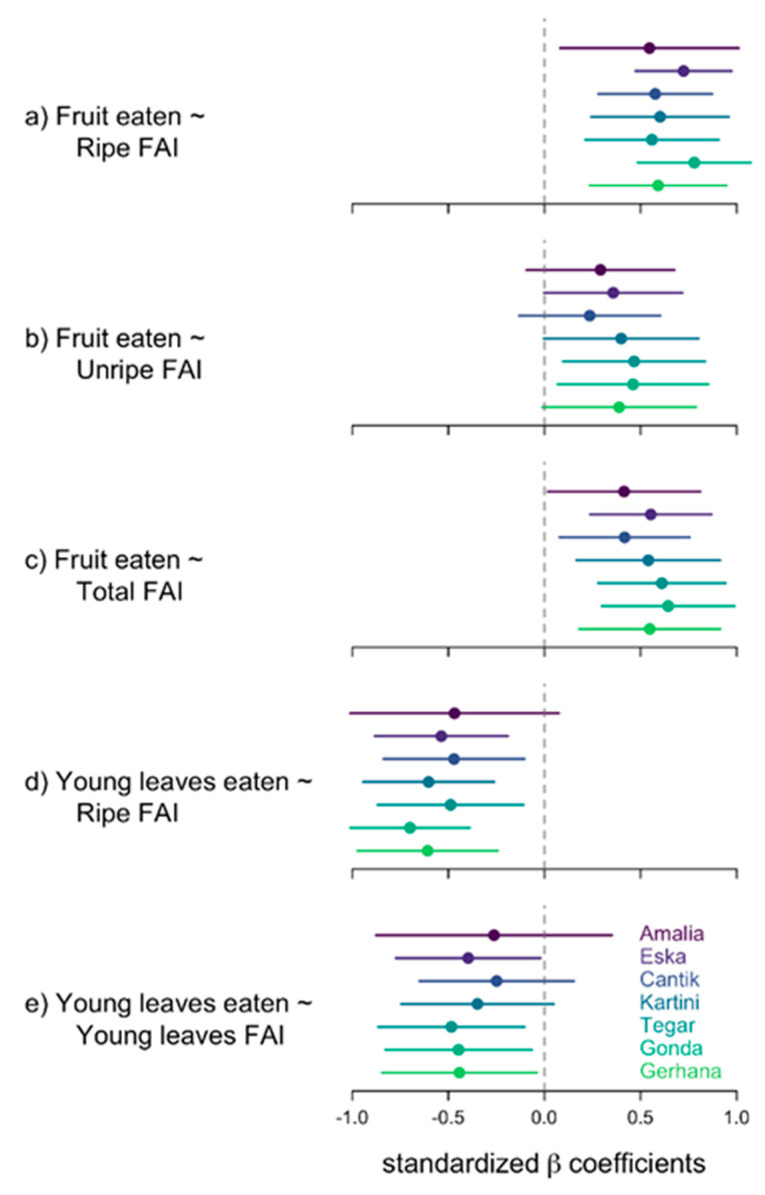
Regression analyses with Fruit Abundance Index (FAI, i.e., percentage of trees fruiting) as predictor and proportion of Fruit eaten out of Forest Food as dependent variable, and with Young Leave Abundance Index (YLAI, i.e., percentage of trees with young leaves) as predictor and proportion of young leaves eaten out of Forest Food as dependent variable. Ripe FAI: only trees with ripe fruit; Unripe FAI: trees with unripe fruit. Total FAI: trees with ripe and unripe fruit; Young LAI: trees with young leaves. Plots show standardized beta coefficients and their 95% confidence intervals.

Phenological conditions varied over the course of our 3-years study period, ranging from very high FAIs (first half) to very low FAIs (second half). We found the abundance of ripe fruit (Ripe FAI) was a reliable predictor of the proportion of fruit in the diet of all orphans (see Figure 7a,c). For most individuals, the availability of (only) unripe fruits (i.e., Unripe FAI) did not predict fruit eaten (Figure 7b), except for two infant males (Tegar and Gonda) (Figure 7b). If ripe fruit was easily available (high FAI) the proportion of young leaves in the orphans’ diet dropped (Figure 7d). The males, but not the females, did eat less young leaves when Young leaves (Young leaves FAI) were abundant (Figure 7e).

**Figure 8:**
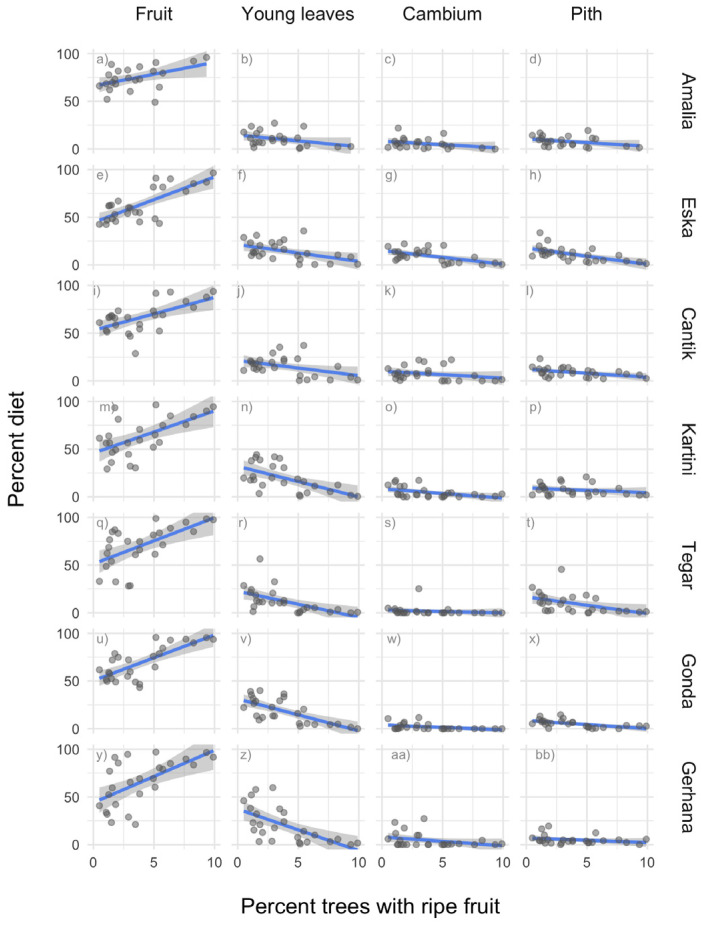
(**a**–**bb**): Scatterplots and regression lines (±95% CI) showing the proportion of plant parts eaten by every orangutan vs. the Ripe Fruit Abundance Index (FAI) between January 2019 to December 2021 (except for Amalia: from August 2019).

We then assessed the interrelations between the proportions of different plant parts in the orphans’ diet. Once the orphans reduced their consumption of fruit, which other parts of plants did they eat more? The correlations between the proportion of fruit and the proportion of young leaves, cambium, and pith, respectively, were highly significantly negative (Table 3). In other words, a decrease in the proportion of fruit eaten was compensated by an increase in the proportion of more fibrous foods. Figure 8a–bb show the proportions of different plant parts eaten per month by each individual. It appears as if the quantity of young leaves in particular is increased when the quantity of fruit is reduced (Figure 8b,f,j,n,r,v,z). Orphans were almost never observed to eat mature leaves, which is in line with findings from wild orangutans [36,38].

Thus, orphans preferred fruit and ate more ripe fruit the more ripe fruit was available. They consumed fewer fibrous plant organs when they ate much fruit, and more when they ate less fruit. Young leaves, pith, and cambium can thus be interpreted as fallback foods.

### 5.4. Did the Orphans Eat Filler Fallback Foods When Fruit Was Scarce?

**Figure 9:**
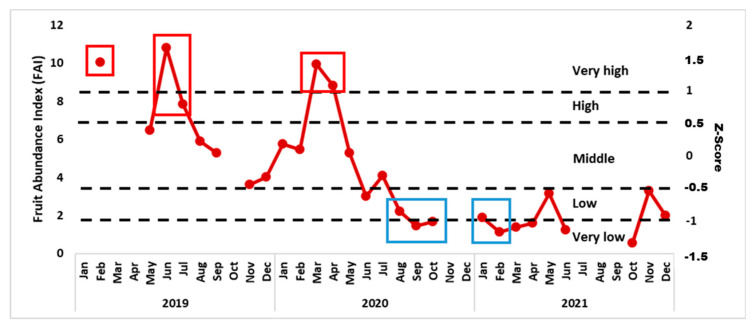
Variation of Ripe FAI in the forest school area between January 2019 and December 2021. Red-framed dots refer to months with a high to very high Ripe FAI, blue-framed dots refer to months with a low to very low Ripe FAI.

Our study period of 3 years covered fruit-abundant and lean periods. To get a sharper focus on how high vs. low fruit availability influenced the food choices of the orphaned orangutans we classified the FAI according to [35] into very low, low, middle, high and very high FAI. Since proportion of Fruit eaten correlated most strongly with the abundance of ripe fruit (Ripe FAI) we chose the five months with a high to very high fruit abundance (February, June and July 2019 as well as March and April 2020) to contrast with the five months of low to very low fruit abundance (August to October 2020 and January to December 2021, see Figure 9). 

**Figure 10:**
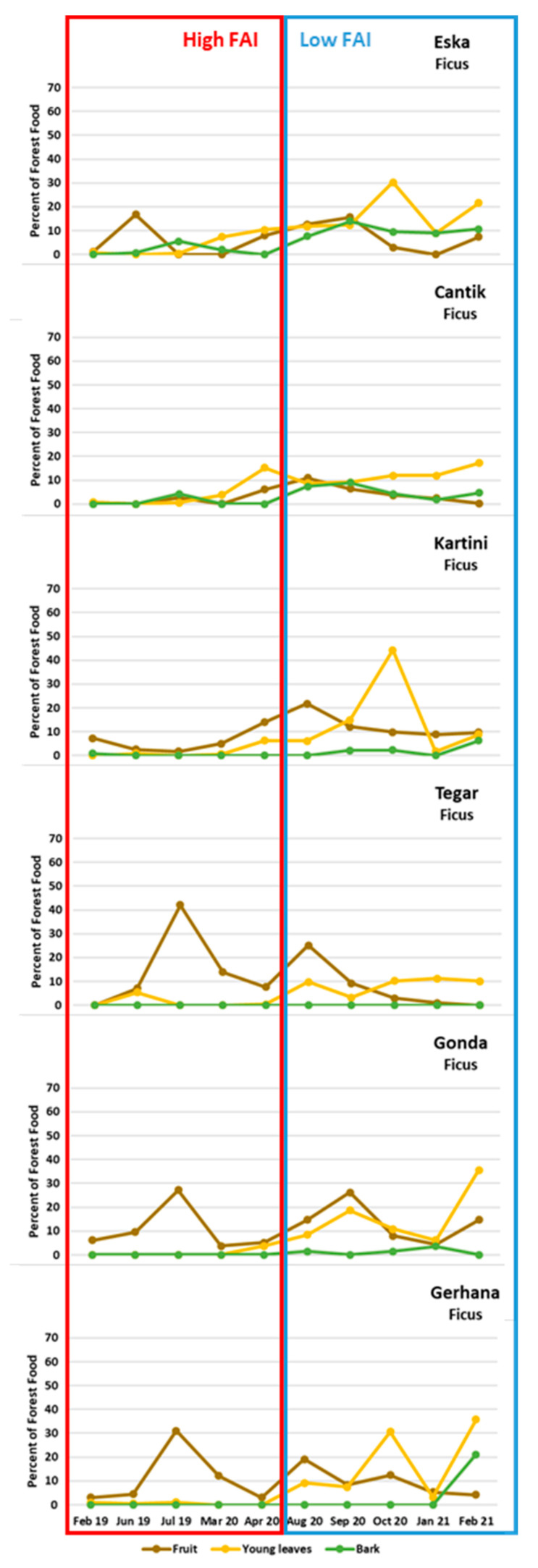
Comparison of parts of *Ficus* spp. eaten during months with a high to very high Ripe FAI vs. months with a low to very low Ripe FAI. Values refer to the respective proportion of plant part eaten (Fruit, Youngs leaves and Bark).

We used this classification to predict whether the parts of plants eaten (i.e., Fruit, Young leaves and Bark) differed between months with High to Very High vs. Low to Very Low Ripe FAI for various genera of *Ficus* spp. We focused on these specific months and not on the whole observation period as data on parts of *Ficus* spp. eaten were only available per orangutan per month and had to be compiled manually. The proportion of Young leaves and Bark of *Ficus* spp. increased during the months with a Low to Very Low Ripe FAI as compared to the months with a High to Very High Ripe FAI (Figure 10). Therefore, young leaves and the bark of *Ficus* spp. could be operationally defined as filler fallback food.

In conclusion, orphans responded to fruit scarcity by eating filler fallback food, especially the young leaves and bark of *Ficus* spp.

### 5.5. Were There Differences in Dietary Breadth? Ad What Caused Them?

**Figure 11:**
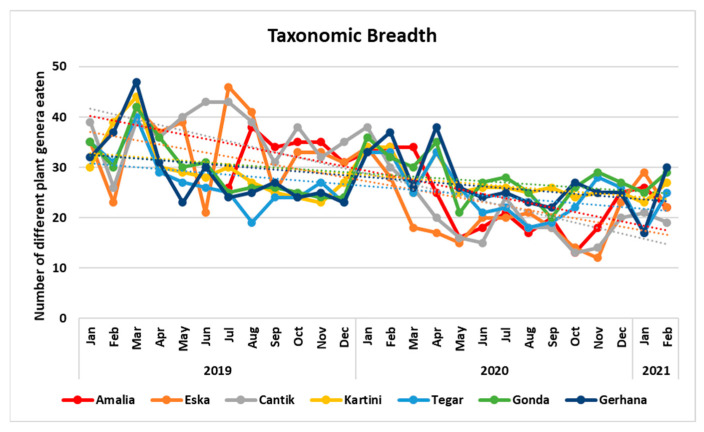
Taxonomic breadth (i.e., number of different plant genera consumed per month). Each line represents one individual.

**Figure 12:**
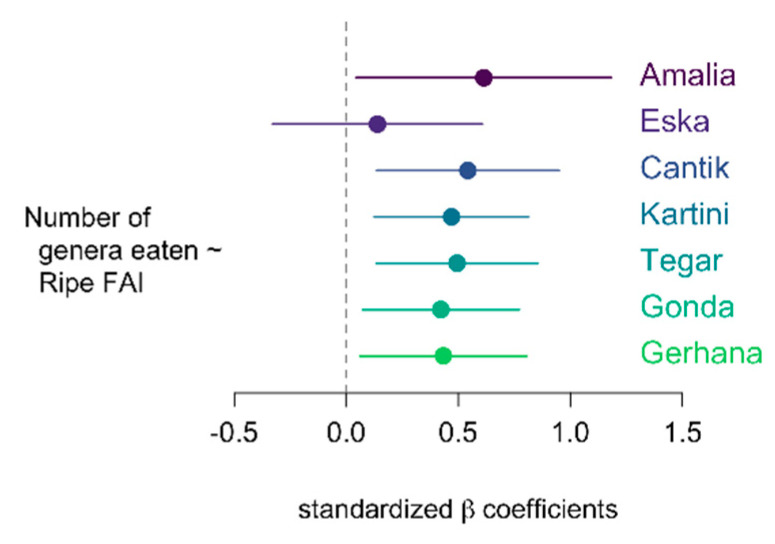
Regression analysis with Ripe Fruit Abundance Index (Ripe FAI, i.e., percentage of trees fruiting) as predictor and number of different genera eaten as dependent variable. Plot shows standardized beta coefficients and their 95% confidence intervals.

One might think that the larger an orangutan’s food repertoire the better s/he would be protected from food scarcity. This would imply that more competent orangutans have larger food repertoires, and possibly also that during periods of fruit scarcity orangutans would evince a larger taxonomic breadth. We tested the latter hypothesis with our data, by correlating the taxonomic breadth of the orphans with the Ripe FAI (Figure 11 and Figure 12). Contrary to our assumption we found that all orphans except of Eska consumed a larger variety of food plant genera during the months of high Ripe FAI. These months also happened to occur at the beginning of the study period, when both the orphans and the caregivers were still new to the area. 

To summarise, orphans ate a larger variety of plants when much fruit was available, but we do not know if all these plants were eaten in the form of fruit. 

## 6. Discussion

We investigated the development of feeding competence in seven orangutan orphans over 3 years while they underwent rehabilitation at a forest school located in old secondary lowland forest in East Kalimantan. The orphans had been confiscated by Indonesian authorities and were directly entrusted to Yayasan Jejak Pulang for rehabilitation with the aim to re-introduce them to their natural habitat once they have “come of age”. It is essential for successful re-introductions that orangutans know where and when to find food, to process it, and what alternatives can be eaten in times of famine. 

In maternally reared wild orangutans, immatures acquire feeding competence mostly by social learning from the mother. By the age of weaning immatures’ diets overlapped on average to 89.5% with that of their mothers [26], with females reaching 100% of their mothers’ diet and males only about 80% [39]. The acquisition of feeding competence in orphans is harder to diagnose. We approached this question via several proxies. 

Question 1: Did the orphans eat what wild orangutans eat?

First, we compared the genera of plants YJP orphans had consumed in their forest school to those free-living orangutans had previously been reported to eat (TOFL, [31,36]). 

Of the plant genera consumed by the orphans, the vast majority (92%) were already known to be orangutan foods [31,36]. Most of these correctly identified foods were genera of trees, but almost one quarter were herb, liana and shrub genera. The remaining 8% of the eaten plants have not been reported to have been consumed by wild orangutans and thus constituted potential mistakes by not-yet competent orphans. The YJP orphans missed out on almost 36% of the 118 edible tree genera known to be available at their forest school. Since those are usually eaten as fruits and only half of these genera did have fruits during the study period, the actual missed opportunities might only be around 20 genera which were available and should have been consumed had the orphans been fully competent. It should be born in mind, however, that an analysis on the level of genera necessarily is more ambiguous than on the level of species.

The YJP orphans’ repertoire of 111 genera is in the middle of the dietary range reported for wild orangutans (21–221 genera) at various study sites [31]. The actual plant genera that were consumed by our orangutans and that were also available in various locations in East Kalimantan overlap by 86.5 to 91.4 percent. The overlap between the undisturbed wild Mentoko population and the orangutans at Jejak Pulang Forest School is 86.5 percent (32 out of the 37 genera available at both sides were consumed by our orangutans). The overlap is only marginally higher between the re-introduced orangutans at Sungai Wain and Meratus, respectively, and our orangutans with 87.7 (57 out of 65 genera) for Sungai Wain and 91.4 percent (53 out of 58 genera) for Meratus.

Question 2: Were there plants which the orangutans ate throughout the year (aka staple foods)?

Using another proxy, we investigated whether the orphans relied on certain plants more heavily, essentially using them as staple foods. It is known from multiple sites that orangutans consume various parts of diverse *Ficus* species year-round [36,40]. Likewise, lianas represent important food sources for orangutans as most do not undergo the mast fruiting rhythms that make fruit of trees so unreliable [15,18,41]. At Tuanan in Central Kalimantan the liana *Leucomphalos callicarpus* is a key resource with fruits, flowers and leaves all being eaten [30].

Five plant genera were eaten by all orphans in over 90% of months of the observation period (Table 2). The most commonly eaten were *Ficus* spp., which grow as climber or as tree. Similarly, *Artabotrys* spp., a liana was eaten in over 90% of the months. This fits the pattern of wild orangutans. The *Borassondendron* palm species was also consumed by YJP orphans throughout the observation period. Among the herbs, *Calamus* spp. (rattan), *Pandanus* spp., *Alpinia* spp. (family Zingiberaceae) and *Bambusa* spp. can be considered staple foods of the YJP orphans. 

It is noteworthy that the plants eaten in less than 85% of the months differ between the juveniles and the weanlings, and that orphans in the same age group had similar preferences. This could be due to social enhancement but also to differences in plant composition between the different areas most frequently visited by the two groups. Age effects are likely to play a role too. For instance, for younger orphans, caregivers would frequently model eating herbs, which is much easier than modelling eating rattan or *Borassodendron borneensis*. *Scleria* spp. (nutrushes, Cyperaceae), a grass-like plant, was eaten frequently by the weanlings but ranked on position 51 for the juveniles.

In sum, the most regularly consumed plants were *Artabotrys* spp. and *Ficus* spp. Also plants that count as herbs and grow either on the forest floor and as climbers were utilized throughout the observation period. This mirrors patterns that are known from wild orangutans at other sites. Traditionally, experts are trained to recognize tree species—and most often economically valuable ones—while liana and herbaceous foods are neglected although they are known to constitute a significant component of orangutan diets at various sites [31] and for YJP orphans as well.

Question 3: Which parts of plants did the orangutan orphans eat, and how did this correspond to their availability in the forest (phenology)? 

Orangutan feeding ecology, particularly for Bornean orangutans, is characterized as “feast or famine”, because the availability of food oscillates so dramatically [16]. The real test of an orangutan’s feeding competence comes when food is scarce and famine looms. From mast to lean periods caloric intake decreases by 24 to 45% (females and males, respectively), a dramatic difference that requires metabolising reserves as indicated by ketones [16]. During masting events fruit is abundant and orangutans gorge themselves on fruit. During lean seasons, orangutans are resorting to eating large quantities of young leaves, pith and cambium, which are therefore often called fallback foods (e.g., [20]).

To assess whether our orphans were able to respond appropriately to food scarcity we had to assess the forest school’s productivity, i.e., analyse phenology data. We used the availability of fruit and young leaves as predictors for the choices between different parts of plants made by the orphans. Phenology data show that the study period started with periods of relatively high fruit abundance in 2019 and in early 2020. Subsequently, the forest was not productive and neither flowers nor fruits occurred in appreciable quantities until 2022. From August 2020, even the production of young leaves was severely reduced.

The orphaned orangutans responded to these variations in similar ways as wild competent orangutans would have done: All consumed large quantities of fruit when ripe fruit was abundant. Some individuals started to increase fruit consumption already while fruit was still unripe. When fruit was not abundant, orangutans increased their intake of pith, cambium and in particular young leaves. 

It is noteworthy that small quantities of pith, cambium and young leaves were eaten throughout, even during periods of fruit abundance. However, although cambium, pith and young leaves were eaten year-round, their proportion in an orphans’ diet did increase when the proportion of fruit decreased, indicating that to some extent these plant parts do serve to compensate for the lack of fruit.

Question 4: Did orphans choose the same fallback foods as wild competent orangutans would have done? 

For *Ficus* spp. the proportion of Young Leaves and Bark increased during the months when ripe fruit was unavailable. Note that, incidentally, the months during which ripe fruit was very scarce were also months of low availability of young leaves. This means that during these months young *Ficus* leaves were “overselected”, i.e., consumed out of proportion with their presence [18]. We therefore conclude that *Ficus,* young leaves in particular, but also cambium serve as filler fallback foods. 

Question 5: Were there differences in taxonomic breadth between individuals, between phenological periods, or within individuals? And what caused them? 

Orphans ate a larger variety of plants when much fruit was available. The number of genera did decline continuously over the study period. This could be in line with Marzec’s finding [12] that re-introduced orangutans narrowed down their dietary variability as they became more familiar with their new home. In our data this trend is confounded with the reduction of fruit availability. We therefore hypothesise that the increased variability of food intake during the fruit-rich periods reflects the same choice pattern that led to wild orangutans’ eating more pulp and seed with increasing fruiting tree diversity at Mentoko ([18], Figure 2b), although our data are inconclusive as to whether all these plants were eaten as fruit. That the orphans did not seem to go through a neophilic experimental phase when they entered forest school, subsequently narrowing down to routinely chosen foods, is corroborated by the orphans taken in after June 2020. This generation of orphans started their term at forest school during the fruit-scarce period and did not achieve the same dietary breadth at the beginning of their forest school tenure as the ones who started in 2018–2019 (YJP, unpubl. data).

How did the orphans know what to eat?

Orangutans conveyed to Yayasan Jejak Pulang for rehabilitation arrive in various conditions and at different ages. Ages of wild orphans are estimated by the pattern of teeth eruption, which has long been considered to be robust and independent of nutritional conditions [42,43]. However, uncertainties remain. How long an orphan has been around people can usually be inferred from their behaviour. Apart from fear and trauma, the orphans also show whether they are already familiar with domesticated plants, with drinking from cups or baby bottles, or simply with being offered food. Some of YJP orphans were totally naïve in all these respects, others were profoundly familiar with human objects and habits. Consequently, the YJP orphans at the forest school differ vastly in their knowledge of forest foods when they first arrive. 

All great apes are particularly dependent on social learning in their acquisition of survival skills (e.g., [44]), corresponding to the extended period of immatures’ dependency on maternal support (e.g., [45]). Observational learning of immatures based on maternal modelling appears to be a key mechanism when young apes develop ecological and social competences. As in other species, the initiative for learning resides primarily in the immature novice ([46], see also [47]). Nonetheless, apes, too, are evolved with instincts, innate preferences and preparedness to learn certain things more readily than others. Tapping into these innate proclivities to create a species-appropriate learning environment can be expected to yield the best results when rehabilitating and educating orphaned orangutans. 

Individual learning of diet items is governed by an evolved, i.e., innate, set of rules because food selection is critical for growth, maintenance, and reproduction [21]. On the proximate level, it is likely that naive food choices are mediated by taste and texture of food items, a mechanism sometimes called “nutritional wisdom” ([48], p. 99). Feedback from the digestive system will determine whether a food choice will be repeated or not (see [32] for an elaboration of this argument). Indeed, acquired taste aversion is famous for being achieved by one-trial learning [49]. In general, natural selection appears to have limited individual learning in the context of food by several mechanisms that safe-guard naïve individuals: taste predilections, one-trial taste aversion learning and copying a bonding partner’s food choices play an important role in avoiding false, potentially lethal choices. In our study, this evolved caution is evident from the proportion of presumed false choices (5%) of tree genera, which is a fraction of the missed opportunities (up to 34%).

At the YJP forest school, rehabilitation is modelled on orangutan child development as documented by field studies (e.g., [45,50], s.a. [51,52], and caregivers are taught how to act similar to orangutan mothers in technical as well as pedagogical ways. An important, and unfortunately largely neglected, aspect is the interplay of attachment quality and the curiosity or exploration motive. In short, securely bonded infants are more explorative and tolerate higher levels of stimulation than are scared infants who lack a secure base [53,54]. 

In the mother-child dyad nutritional learning begins with the eruption of the first teeth, mostly by sucking and chewing on food items that the mother is occupied with. Infants familiarize themselves with the smell and view of maternal foods, and chew on discarded parts. Rarely, a mother presents food to her child, but immatures often attempt to obtain chewed or fresh foods from their mothers. Human infants, too, identify intimacy in a relationship with the sharing of saliva, i.e., food sharing [55]. Observational learning is ubiquitous, and immatures pay great attention to what mothers do with foods, while mothers are modelling inadvertently, as they pursue their daily activities.

**Figure 13:**
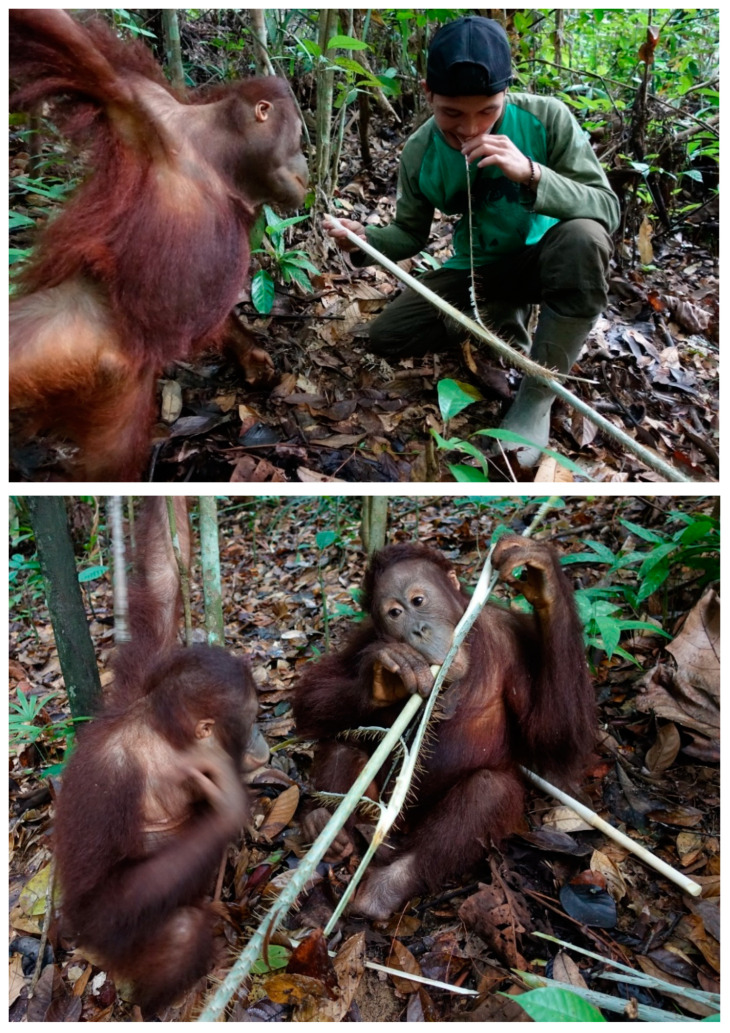
(**Above**) Caregiver Nor Faniyansah models peeling off the spiny skin of a rattan shoot to 6-year-old Eska. (**Below**) Later, Eska is peered at by 5-year-old Cantik as he consumes rattan pith. Cantik was eventually permitted to eat fallen parts of rattan pith (not shown).

Human surrogate mothers are far less proficient in identifying and obtaining orangutan foods, because they lack knowledge and do not routinely climb into the canopy. Instead of following their mothers finding food in the canopy, orphans have learned to follow point and climb up for harvesting while their surrogate mothers wait down below. Caregivers at YJP play an active role in creating opportunities for orangutans to learn about food. Moreover, they facilitate and coach orphans’ learning of such food items as suitable for orangutan consumption, thus limiting the risks of random and uninformed individual experimentation (Figure 13).

In sum, the immersion of the orphans in a natural forest environment, the presence of conspecifics as role models and competitors, and directions and modelling by the caregivers all coalesce to create the learning environment of the orphans. Social facilitation, social enhancement, modelling and individual experimentation are combined. It is likely that quite unwittingly the orphans and caregivers develop a particular YJP food culture that will probably emerge more clearly once orangutans are re-homed to the release site. 

## Figures and Tables

**Table 1:** Biographic information on subjects in the forest school.

Name	Sex	Year of Intake	Est. Age at Intake	Month of Entry FS	Est. Age at Onset of Study Period	Age Group	Forest School Level	Study Period
Amalia	Female	2017	6 yrs	Jul 2019	8 yrs	juvenile	FS3	Aug 2019–Dec 2021
Eska	Male	2017	4 yrs	Nov 2017	6 yrs	juvenile	FS2/3	Jan 2019–Dec 2021
Cantik	Female	2017	3 yrs	Oct 2017	5 yrs	juvenile	FS2/3	Jan 2019–Dec 2021
Kartini	Female	2018	17+ mo	May 2018	>2 yrs	weanling	FS1/2	Jan 2019–Dec 2021
Tegar	Male	2017	12 mo	Oct 2017	27 mo	weanling	FS1/2	Jan 2019–Dec 2021
Gonda	Male	2017	8 mo	Oct 2017	25 mo	weanling	FS1/2	Jan 2019–Dec 2021
Gerhana	Male	2018	9 mo	May 2018	19 mo	weanling	FS1/2	Jan 2019–Dec 2021

**Table 2:** Plant genera eaten in at least 50 percent of months by the seven individuals in the Forest school between January 2019 and February 2021.

Plant Genus	Growth Form	Percent of Months Eaten		Frequency Rank	
		Mean ± SD	Range	Juveniles	Weanlings
*Ficus* spp.	Tree, Liana	99 ± 2	95–100	1	1
*Artabotrys* spp.	Liana	98 ± 2	96–100	4	2
*Calamus* spp.	Herb	98 ± 2	95–100	1	3
*Borassodendron* spp.	Tree	96 ± 4	92–100	1	5
*Alpinia* spp.	Herb	91 ± 12	65–100	6	4
*Artocarpus* spp.	Tree	85 ± 5	81–92	5	10
*Pandanus* spp.	Herb	85 ± 8	75–92	8	8
*Pternandra* spp.	Tree	83 ± 10	65–92	10	7
*Bambusa* spp.	Herb	80 ± 19	45–96	14	6
*Callicarpa* spp. *	Tree	78 ± 10	62–88	11	9
*Syzygium* spp.	Tree	75 ± 9	62–85	7	14
*Diospyros* spp.	Tree	66 ± 26	27–88	8	19
*Fordia* spp.	Tree	59 ± 21	25–85	21	12
*Dracontomelon* spp.	Tree	57 ± 19	31–77	22	13
*Maesa* spp.	Liana	57 ± 11	38–73	12	20
*Oncosperma* spp.	Tree	55 ± 8	46–65	13	22
*Scleria* spp.	Herb	52 ± 31	12–81	50	11
*Macaranga* spp.	Tree	51 ± 10	38–65	16	21

* *Callicarpa* spp. is not found in the orangutan food list (TOFL) but in [36].

**Table 3:** Pearson correlation of share of Fruit and Young leaves, Cambium, and Pith. All food items are share of total Forest Food.

Forest Food	Amalia	Eska	Cantik	Kartini	Tegar	Gonda	Gerhana
Fruit—Young leaves	r = −0.544 ***p* = 0.003**	r = −0.756 ***p* < 0.001**	r = −0.822 ***p* < 0.001**	r = −0.872 ***p* < 0.001**	r = −0.758 ***p* < 0.001**	r = −0.846 ***p* < 0.001**	r = −0.843 ***p* < 0.001**
Fruit—Cambium	r = −0.641 ***p* < 0.001**	r = −0.720 ***p* < 0.001**	r = −0.614 ***p* < 0.001**	r = −0.481 ***p* = 0.003**	r = −0.478 ***p* = 0.003**	r = −0.316 *p* = 0.065	r = −0.682 ***p* < 0.001**
Fruit—Pith	r = −0.720 ***p* < 0.001**	r = −0.710 ***p* < 0.001**	r = −0.730 ***p* < 0.001**	r = −0.495 ***p* = 0.003**	r = −0.703 ***p* < 0.001**	r = −0.552 ***p* = 0.001**	r = −0.313 *p* = 0.063

*p*-values < 0.05 are shown in bold.

## Data Availability

Data are reported in the Results and Supplementary Materials section of this paper. Upon request, additional information can be obtained from the corresponding authors.

## Supplementary Materials

The following supporting information can be downloaded at: https://www.mdpi.com/article/10.3390/ani13132111/s1, Table S1: YJP list of plants eaten over 26 months from January 2019 to February 2021; Table S2: Food trees present in the forest school and producing fruit during the observation period; Table S3: Relative frequencies (i.e., percent of months) per individual of the plant genera eaten most frequently; Table S4: YJP food plant list including genera and species
