# Supplementary material for: The Development of Feeding Competence in Rehabilitant Orphaned Orangutans and How to Measure It
Source: Animals (Basel). 2023 Jun 26;13(13):2111. doi: 10.3390/ani13132111 (PMC10339905; doi:10.3390/ani13132111)
Supplement: Supplementary file 1 [file animals-13-02111-s001.zip › animals-2412084-supplementary.pdf]

Supplementary Materials

The development of feeding competence in rehabilitant orphaned orangutans and how to measure it

Signe Preuschoft, Andrew J. Marshall, Lorna Scott, Siti Nur Badriyah, Melki Deus T. Purba, Erma Yuliani, Paloma Corbi, Ishak Yassir, M. Ari Wibawanto, Elfriede Kalcher-Sommersguter

Table S1: YJP list of plants eaten over 26 months from January 2019 to February 2021.

**Family**	**Genus**	**Growth form**	**Parts of Plants consumed**	**Mean** (% of months consumed)	**Range** (% of months consumed)	**In plant lists****	**Modelled by caregivers**
Moraceae	*Ficus* spp	Tree, Liana	B, F, L, St, YL	99	95-100	Yes	Yes
Annonaceae	*Artabotrys* spp	Liana	F	98	96-100	Yes	No
Aracaceae	*Calamus* spp	Herb (Rattan)	F, P	98	95-100	Yes	Yes
Aracaceae	*Borassodendron* spp	Tree (Palm)	F, Fl, P	96	92-100	Yes	Yes
Zingiberaceae	*Alpinia* spp	Herb	F, P	91	65-100	Yes	Yes
Moraceae	*Artocarpus* spp	Tree	B, F, St, YL	85	81-92	Yes	Yes
Pandanaceae	*Pandanus* spp	Herb	F, P	85	75-92	Yes	Yes
Melastomataceae	*Pternandra* spp	Tree	F	83	65-92	Yes	Yes
Gramineae	*Bambusa* spp	Herb	P	80	45-96	Yes	Yes
Lamiaceae	*Callicarpa* spp	Tree	F, Fl	78	62-88	Yes	Yes
Myrtaceae	*Syzygium* spp	Tree	F	75	62-85	Yes	Yes
Ebenaceae	*Diospyros* spp	Tree	F	66	27-88	Yes	Yes
Leguminosae	*Fordia* spp	Tree	Fl, S, St	59	25-85	Yes	Yes
Anacardiaceae	*Dracontomelon* spp	Tree	F	57	31-77	Yes	No
Myrsinaceae	*Maesa* spp	Liana	F	57	38-73	Yes	No
Arecaceae	*Oncosperma* spp	Tree (Palm)	F, Fl, P	55	46-65	Yes	Yes
Cyperaceae	*Scleria* spp	Herb	St	52	12-81	Yes	Yes
Euphorbiaceae	*Macaranga* spp	Tree	S	51	38-65	Yes	Yes
Annonaceae	*Polyalthia* spp	Tree	F	48	38-65	Yes	Yes
Marantaceae	*Donax* spp	Herb	St	46	5-81	Yes	No
Dilleniaceae	*Dillenia* spp	Tree	F, Fl, St	43	27-65	Yes	Yes
Hypoxidaceae	*Curculigo* spp	Herb	F, P	42	10-54	Yes	Yes
Sterculiaceae	*Sterculia* spp	Tree	Fl, S	38	0-69	Yes	Yes
Pteridophyta	*Stenochlaena* spp	Herb, Climber	St, YL	37	0-69	Yes	Yes
Rubiaceae	*Urophyllum* spp	Tree	F	36	20-54	Yes	No
Vitaceae	*Leea* spp	Tree	F, St	36	0-62	Yes	No
Fabaceae	*Archidendron* spp	Tree	B, S	35	23-54	Yes	Yes
Polygalaceae	*Xanthophyllum* spp	Tree	F, Fl, L, St, YL	34	15-60	Yes	Yes
Euphorbiaceae	*Baccaurea* spp	Tree	F, Fl	32	27-38	Yes	Yes
Myristicaceae	*Knema* spp	Tree	F	32	15-60	Yes	Yes
Euphorbiaceae	*Aporosa* spp	Tree	F	32	27-40	Yes	Yes
Irvingiaceae	*Irvingia* spp	Tree	F	31	23-42	Yes	Yes
Annonaceae	*Cananga* spp	Tree	F	31	0-50	No	No
Clusiaceae	*Garcinia* spp	Tree	F	30	23-42	Yes	Yes
Ulmaceae	*Gironniera* spp	Tree	B, F	30	12-46	Yes	No
Fagaceae	*Castanopsis* spp	Tree	S	30	23-35	Yes	Yes
Lauraceae	*Litsea* spp	Tree	F	30	23-35	Yes	No
Rutaceae	*Melicope* spp	Tree	B, S	29	15-35	No	Yes
Symplocaceae	*Symplocos* spp	Tree	F, Fl, YL	29	0-70	Yes	No
Sapotaceae	*Palaquium* spp	Tree	F	28	15-38	Yes	No
Dracaenaceae	*Dracaena* spp	Herb	F, P	26	12-50	Yes	No
Euphorbiacea	*Antidesma* spp	Tree	F	24	12-42	Yes	Yes
Melastomataceae	*Clidemia* spp	Shrub	F	24	5-42	Yes	Yes
Elaeocarpaceae	*Elaeocarpus* spp	Tree	F	24	15-35	Yes	Yes
Athyriaceae	*Diplazium* spp	Herb	St	23	0-38	No	Yes
Poaceae	*Saccharum* spp	Herb	St	21	0-38	No	Yes
Fagaceae	*Lithocarpus* spp	Tree	S	20	15-27	Yes	Yes
Sapotaceae	*Madhuca* spp	Tree	F	19	15-27	Yes	No
Lecythidaceae	*Barringtonia* spp	Tree	Fl, S	18	4-35	Yes	Yes
Aracaceae	*Pholidocarpus* spp	Tree (Palm)	F	18	8-38	Yes	No
Passifloraceae	*Adenia* spp	Liana	F, S	16	0-27	Yes	No
Euphorbiaceae	*Bridelia* spp	Tree	F	15	10-19	Yes	Yes
Meliaceae	*Aglaia* spp	Tree	F, S	14	8-20	Yes	Yes
Alangiaceae	*Alangium* spp	Tree	F, S	14	12-20	Yes	Yes
Euphorbiaceae	*Glochidion* spp	Tree	F	14	4-35	Yes	No

Family	Genus	Growth form		Mean* (% of months consumed)	Range* (% of months consumed)	In plant lists**	Modelled by caregivers
Fabaceae	*Spatholobus* spp	Liana	S	14	4-45	Yes	Yes
Sapindaceae	*Nephelium* spp	Tree	F	14	8-20	Yes	Yes
Smilacaceae	*Smilax* spp	Liana	F, St	14	0-35	Yes	No
Asteraceae	*Vernonia* spp	Tree	B, F, Fl	14	0-31	Yes	Yes
Rubiaceae	*Ixora* spp	Shrub	F, Fl	14	4-30	Yes	Yes
Rubiaceae	*Neonauclea* spp	Tree	F	13	4-27	Yes	No
Cyperaceae	*Mapania* spp	Herb	F, P	13	0-23	Yes	No
Combretaceae	*Terminalia* spp	Tree	S	13	0-31	Yes	No
Apocynaceae	*Alstonia* spp	Tree	B	12	0-35	Yes	Yes
Zingiberaceae	*Zingiber* spp	Herb	F, P	11	5-15	Yes	Yes
Lamiaceae	*Vitex* spp	Tree	F	11	4-15	Yes	No
Myrsinaceae	*Embelia* spp	Liana	F	10	0-30	Yes	Yes
Bombacaceae	*Durio* spp	Tree	F	10	4-20	Yes	No
Lecythidaceae	*Planchonia* spp	Tree	F	10	0-23	Yes	No
Lauraceae	*Alseodaphne* spp	Tree	F	9	0-19	Yes	No
Lauraceae	*Eusideroxylon* spp	Tree	F	9	0-25	Yes	No
Melastomataceae	*Melastoma* spp	Tree	F, Fl	9	5-19	Yes	Yes
Euphorbiaceae	*Mallotus* spp	Tree	S	9	0-31	Yes	No
Annonaceae	*Popowia* spp	Tree	F	8	4-12	Yes	Yes
Tiliaceae	*Microcos* spp	Tree	F	8	0-25	Yes	No
Ampelidaceae	*Tetrastigma* spp	Liana	F	8	0-15	Yes	No
Fabaceae	*Callerya* spp	Liana	F, Fl, S	7	4-12	Yes	Yes
Burseraceae	*Dacryodes* spp	Tree	F	7	4-12	Yes	Yes
Euphorbiaceae	*Paracroton* spp	Tree	F, Fl	6	0-15	No	No
Meliaceae	*Reinwardtiodendron* spp	Tree	F	6	4-10	Yes	No
Myrsinaceae	*Ardisia* spp	Tree	F, Fl	6	4-15	Yes	No
Actinidiaceae	*Saurauia* spp	Tree	F	6	0-15	Yes	No
Zingiberaceae	*Plagiostachys* spp	Herb	F, St	5	0-12	Yes	No
Sapindaceae	*Dimocarpus* spp	Tree	F	3	0-4	Yes	No
Cornaceae	*Mastixia* spp	Tree	F	3	0-10	Yes	Yes
Anacardiaceae	*Mangifera* spp	Tree	F	3	0-10	Yes	No
Myrtaceae	*Rhodamnia* spp	Tree	F	3	0-10	Yes	No
Olacaceae	*Scorodocarpus* spp	Tree	F	3	0-8	Yes	No
Annonaceae	*Goniothalamus* spp	Tree	F	3	0-8	Yes	No
Rubiaceae	*Porterandia* spp	Tree	F	3	0-8	Yes	No
Bombacaceae	*Neesia* spp	Tree	S	3	0-10	Yes	No
Euphorbiaceae	*Cleistanthus* spp	Tree	F	2	0-8	Yes	No
Myristicaceae	*Myristica* spp	Tree	F	2	0-5	Yes	No
Dipterocarpaceae	*Dipterocarpus* spp	Tree	S	2	0-8	Yes	No
Meliaceae	*Lansium* spp	Tree	F	2	0-8	Yes	No
Thymelaeaceae	*Aquilaria* spp	Tree	B, F	2	0-5	Yes	No
Sapindaceae	*Guioa* spp	Tree	F	2	0-5	No	No
Araceae	*Aglaonema* spp	Herb	F	2	0-4	Yes	No
Lauraceae	*Cryptocarya* spp	Tree	F	1	0-5	Yes	No
Burseraceae	*Canarium* spp	Tree	F	1	0-4	Yes	No
Connaraceae	*Cnestis* spp	Liana	F	1	0-4	Yes	No
Hypericaceae	*Cratoxylum* spp	Tree	F	1	0-8	Yes	No
Fabaceae	*Cynometra* spp	Tree	F	1	0-4	Yes	No
Lauraceae	*Caryodaphnopsis* spp	Tree	S	1	0-4	No	No
Annonaceae	*Enicosanthum* spp	Tree	F	1	0-4	Yes	No
Myristicaceae	*Horsfieldia* spp	Tree	F	1	0-4	Yes	No
Dilleniaceae	*Tetracera* spp	Liana	F	1	0-5	Yes	No
Theaceae	*Camellia* spp	Tree	F	1	0-4	No	No
Dipterocarpaceae	*Shorea* spp	Tree	S	1	0-4	Yes	No
Annonaceae	*Phaenthus* spp	Tree	F	1	0-4	No	No
Apocynaceae	*Willughbeia* spp	Liana	F	1	0-4	Yes	No

*Mean and range over all 7 orangutans (i.e., over 26 months for Eska, Cantik, Kartini, Tegar, Gonda and Gerhana; over 20 months for Amalia); **Russon et al. 2009 and/or Kanamori et al. 2010

Abbreviations: B = Bark, F = Fruit, Fl = Flower, L = Leaves, P = Pith, S= Seed, St = Stem, YL= Young Leaves

Table S2: Food trees present in the forest school and producing fruit during the observation period.

Missed opportunities	Month(s) of fruiting	
	**2019**	**2020**
Callophyllum spp	Sep, Nov, Dec	
Cotylelobium spp	Jun, Jul, Dec	Jan
Croton spp	Nov	
Duabanga spp	Feb, Jul, Dec	Jun, Jul
Endospermum spp	Jun, Jul, Dec	
Gluta spp	Nov, Dec	Jan, Mar
Gonystylus spp	Dec	
Gymnacranthera spp	Nov, Dec	
Hopea spp	Dec	Jan, Feb
Hydnocarpus spp	Nov, Dec	Jan, Feb
Ilex spp		Jan
Koompassia spp		Jan
Magnolia spp	Feb, Nov	Feb
Monocarpia spp		Jan
Ochanostachys spp		Jan
Payena spp		Apr, May
Pometia spp	Feb	
Ryparosa spp	Feb, May	
Schima spp	Jun, Jul, Dec	Jan, Feb, Aug, Sep
Vatica spp		Sep

Table S3: Relative frequencies (i.e., percent of months) per individual of the plant genera eaten most frequently.

**Plant genera**	**Amalia**	**Eska**	**Cantik**	**Kartini**	**Tegar**	**Gonda**	**Gerhana**
Borassodendron sp	95	100	100	92	92	92	100
Ficus sp	95	100	100	100	100	100	100
Calamus sp	95	100	100	96	100	100	96
Artocarpus sp	90	92	81	88	81	85	81
Alpinia sp	65	96	92	100	88	96	96
Syzygium sp	85	85	77	65	77	77	62
Pandanus sp	75	77	88	92	92	92	77
Diospyros sp	75	77	88	27	31	77	85
Pternandra sp	65	73	85	92	88	92	85
Callicarpa sp	80	62	65	88	81	85	85
Bambusa sp	45	69	69	92	96	92	92
Fordia sp	25	46	46	73	62	85	77
Scleria sp	20	12	27	69	73	81	81
Artabotrys sp	100	96	96	100	100	100	96
Donax sp	5	27	15	81	69	69	58

Table S4: YJP food plant list including genera and species.

Genus	Species	Part of plant
Adenia spp	Adenia macrophylla	
Aglaia spp	Aglaia macrocarpa	
Aglaonema spp		
Alangium spp	Alangium javanicum	
	Alangium longiflorum	
	Alangium ridley	F
Alpinia spp		
Alseodaphne spp	Alseodaphne elmerii	
Alstonia spp	Alstonia iwahigensis	Sa
	Alstonia scholaris	F
Antidesma spp	Antidesma neurocarpum	
Aporosa spp	Aporosa frutescens	
	Aporosa grandistipula	
	Aporosa indica	
	Aporosa lucida	
	Aporosa nitida	F, S
	Aporosa subcaudata	S
Aquilaria spp	Aquilaria beccariana	
	Aquilaria microcarpa	F
Archidendron spp	Archidendron clypearia	
	Archidendron jiringa	
	Archidendron microcarpum	YL
Ardisia spp	Ardisia crenata	
	Ardisia forbesii	
Artabotrys spp	Artabotrys anisophyllus	
	Artabotrys hexapetalus	
	Artabotrys suaveolens	
Artocarpus spp	Artocarpus anisophyllus	F, YL, Sa, Fl, S
	Artocarpus dadah	F, YL
	Artocarpus elasticus	F, Sa
	Artocarpus integer	F, YL
	Artocarpus kemando	
	Artocarpus lanceifolius	F, YL, Sa
	Artocarpus nitidus	F
	Artocarpus odoratissimus	F, L, B
	Artocarpus rigidus	F, Sa
	Artocarpus tamaran	F, YL
	Artocarpus tomentosulus	
Baccaurea spp	Baccaurea lanceolata	F
	Baccaurea macrocarpa	F
	Baccaurea odoratissima	
	Baccaurea parviflora	
	Baccaurea tetrandra	

Genus	Species	Part of plant
Bambusa spp		
Barringtonia spp	Barringtonia macrostachya	
Borassodendron spp	Borassodendron borneensis	F, Fl, P
Bridelia spp	Bridelia glauca	F
Calamus spp	Calamus manan	
Callerya spp		
Callicarpa spp	Callicarpa longifolia	
	Callicarpa pentandra	
Calophyllum spp	Calophyllum nodosum	F
Camellia spp		
Cananga spp	Cananga odorata	F, B
Canarium spp	Canarium littorale	F
Caryodaphnopsis spp	Caryodaphnopsis tonkinensis	
Castanopsis spp	Castanopsis cf evansii	
	Castanopsis fulva	F
Cleistanthus spp	Cleistanthus myrianthus	
Clidemia spp	Clidemia hirta	
Cnestis spp		
Cotylelobium spp	Cotylelobium melanoxylon	S
Cratoxylum spp	Cratoxylum sumatranum	
Croton spp		
Cryptocarya spp	Cryptocarya densiflora	
Curculigo spp	Curculigo latifolia	
Cynometra spp		
Dacryodes spp	Dacryodes costata	
	Dacryodes rostrata	F
	Dacryodes rugosa	F
Dillenia spp	Dillenia excelsa	F, Fl
	Dillenia indica	
	Dillenia nervosa	
	Dillenia reticulata	F
	Dillenia suffruticosa	
Dimocarpus spp	Dimocarpus longan	F
Diospyros spp	Diospyros borneensis	F, YL
	Diospyros confertiflora	F, S, YL
	Diospyros macrophylla	
	Diospyros cf sumatrana	
Diplazium spp	Diplazium esculentum	
Dipterocarpus spp	Dipterocarpus convertus	
	Dipterocarpus cornutus	S, Fl
	Dipterocarpus humeratus	S, L, B
	Dipterocarpus tempehes	S
Donax spp	Donax canniformis	
Dracaena spp		
Dracontomelon spp	Dracontomelon dao	F, YL, P

Genus	Species	Part of plant
Duabanga spp	Duabanga moluccana	
Durio spp	Durio acutifolius	F, S
	Durio dulcis	F, S
	Durio excelsus	
	Durio graveolens	
	Durio lanceolatus	F
	Durio zibethinus	
Elaeocarpus spp	Elaeocarpus macrocerus	
	Elaeocarpus stipularis	F, S
	Elaeocarpus valetonii	
Embelia spp	Embelia javanica	
Endospermum spp	Endospermum diadenum	YL
Enicosanthum spp	Enicosanthum paradoxum	Fl
Eusideroxylon spp	Eusideroxylon zwageri	F, YL, Fl
Ficus spp	Ficus aurata	
	Ficus callophylla	
	Ficus grossularioides	F, YL
	Ficus heteropleura	
	Ficus parviflora	
	Ficus punctata	
	Ficus stricta	
	Ficus sp 1	
	Ficus sp 2	
	Ficus sp 3	
	Ficus variegata	F, B
Fordia spp	Fordia splendidissima	F, YL
Garcinia spp	Garcinia parvifolia	F, L, YL
Gironniera spp	Gironniera nervosa	F, YL, Sa
Glochidion spp	Glochidion arborescens	
	Glochidion sericeum	
Gluta spp	Gluta macrocarpa	
	Gluta wallichii	F, YL, Sa, Fl
Goniothalamus spp	Goniothalamus macrophyllus	
Gonystylus spp	Gonystylus affinis	F
	Gonystylus qansanguenius	
Guioa spp	Guio pleueropteris	
Gymnacranthera spp	Gymnacranthera farquhariana	F, B
Hopea spp	Hopea mangarawan	
	Hopea rudiformis	
Horsfieldia spp	Horsfieldia grandis	F
Hydnocarpus spp	Hydnocarpus polypetala	
Ilex spp	Ilex cymosa	F, S, YL
Irvingia spp	Irvingia malayana	F, Sa
Ixora spp	Ixora caudata	
	Ixora parviflora	

Genus	Species	Part of plant
Knema spp	Knema latericia	F
	Knema latifolia	
	Knema laurina	
	Knema pallens	
	Kenam pulchra	
Koompassia spp	Koompassia malaccensis	L
Lansium spp		
Leea spp	Leea aculeata	
	Leea indica	F
Lithocarpus spp	Lithocarpus blumcanus	
	Lithocarpus coopertus	F
	Lithocarpus gracilis	F
Litsea spp	Litsea cf angulata	
	Litsea elliptica	
	Litsea firma	
	Litsea garciae	F, YL
Macaranga spp	Macaranga bancana	
	Macaranga conifera	F
	Macaranga gigantea	F
	Macaranga hypoleuca	F
	Macaranga motleyana	
	Macaranga pearsonii	B
	Macaranga tanarius	
Madhuca spp	Madhuca motleyana	F, Sa, Fl
	Madhuca pierrei	
	Madhuca sericea	F, YL
Maesa spp	Maesa ramentacea	
Magnolia spp		
Mallotus spp	Mallotus nesophilus	
	Mallotus penangensis	
Mangifera spp	Mangifera foetida	F
Mapania spp		
Mastixia spp		
Melastoma spp	Melastoma malabathricum	
Melicope spp	Melicope glabra	F
Microcos spp	Microcos antidesmifolia	
	Microcos crassifolia	F, Sa
	Microcos tomentosa	F, YL
Monocarpia spp	Monocarpia kalimantanensis	
Myristica spp	Myristica elliptica	
	Myristica iners	
	Myristica maxima	F
Neesia spp	Neesia synandra	S
Neonauclea spp	Neonauclea gigantea	
Nephelium spp	Nephelium cuspidatum	F

Genus	Species	Part of plant
Nephelium spp	Nephelium lappaceum	F, S
	Nephelium ramboutan-ake	F
Ochanostachys spp	Ochanostachys amentacea	
Oncosperma spp	Oncosperma horridum	P
	Oncosperma tigillarium	
Palaquium spp	Palaquium beccarianum	
	Palaquium quercifolium	F, Sa
	Palaquium rostratum	F
	Palaquium sericeum	F, YL
	Palaquium stenophyllum	F
Pandanus spp		
Paracroton spp	Paracroton pendulus	
Payena spp	Payena lucida	F
Phaenthus spp	Phaeantus splendens	
Pholidocarpus spp	Pholidocarpus kingianus	
Plagiostachys spp		
Planchonia spp		
Polyalthia spp	Polyalthia cauliflora	
	Polyalthia lateriflora	
	Polyalthia rumphii	F
	Polyalthia sumatrana	
Pometia spp	Pometia pinnata	F, S, B
Popowia spp	Popowia hirta	
	Popowia pisocarpa	
Porterandia spp	Porterandia anisophylla	F, YL
Pternandra spp	Pternandra azurea	
	Pternandra coerulescens	
	Pternandra galeata	
	Pternandra rostrata	
Reinwardtiodendron spp		
Rhodamnia spp	Rhodamnia cinerea	F
Ryparosa spp	Ryparosa kostermansii	
Saccharum spp	Saccharum spontaneum	
Saurauia spp	Saurauia leucocarpa	
Schima spp	Schima wallichii	B
Scleria spp	Scleria sumatrensis	
	Scleria terrestris	
Scorodocarpus spp	Scorodocarpus borneensis	F
Shorea spp	Shorea johorensis	
	Shorea laevis	S
	Shorea lamelata	
	Shorea leprosula	L, B
	Shorea ovalis	S
	Shorea parvifolia	S
	Shorea pauciflora	

Genus	Species	Part of plant
Shorea	Shorea seminis	
	Shorea smithiana	
Smilax spp		
Spatholobus spp	Spatholobus ferrugineus	
Stenochlaena spp	Stenochlaena palustris	
Sterculia spp	Sterculia lanceifolia	
	Sterculia macrophylla	S, B
	Sterculia rubiginosa	YL
	Sterculia cf. stipulata	YL
Symplocos spp	Symplocos fasciculata	F, L, YL
Syzygium spp	Syzygium claviflorum	
	Syzygium lineatum	F
	Syzygium tawahense	F
	Syzygium zeylanicum	
Terminalia spp	Terminalia catappa	
	Terminallia foetidissima	F
	Terminalia pallida	
Tetracera spp		
Tetrastigma spp		
Urophyllum spp	Urophyllum arboreum	
Vatica spp	Vatica umbonata	
	Vatica vinosa	
Vernonia spp	Vernonia arborea	B
Vitex spp	Vitex pinnata	L, YL
Willughbeia spp	Willughbeia angustifolia	
Xanthophyllum spp	Xanthophyllum affine	
	Xanthophyllum obscurum	F, L, YL
Zingiber spp	Zingiber acaea	

Abbreviations: B = Bark, F = Fruit, Fl = Flower, L = Leaves, P = Pith, S= Seed, Sa = Sap, YL= Young Leaves

Red = missed opportunities (see text for explanation)
Blue = possible mistakes (see text for explanation)
